# “What if that’s your last sleep?” A qualitative exploration of the trauma of incarceration and sleep

**DOI:** 10.1093/sleepadvances/zpad055

**Published:** 2023-12-27

**Authors:** Johanna E Elumn, Patrick Li, Malcolm S Lytell, Marisol Garcia, Emily A Wang, Henry Klar Yaggi

**Affiliations:** SEICHE Center for Health and Justice, Yale School of Medicine, New Haven, CT, USA; Section of General Internal Medicine, Yale School of Medicine, New Haven, CT, USA; SEICHE Center for Health and Justice, Yale School of Medicine, New Haven, CT, USA; Section of General Internal Medicine, Yale School of Medicine, New Haven, CT, USA; SEICHE Center for Health and Justice, Yale School of Medicine, New Haven, CT, USA; Section of General Internal Medicine, Yale School of Medicine, New Haven, CT, USA; Vermont Law School, South Royalton, VT, USA; SEICHE Center for Health and Justice, Yale School of Medicine, New Haven, CT, USA; Section of General Internal Medicine, Yale School of Medicine, New Haven, CT, USA; Clinical Epidemiology Research Center, VA CT HCS, West Haven CT, USA; Section Pulmonary, Critical Care and Sleep Medicine, Department of Internal Medicine, Yale University School of Medicine, New Haven, CT, USA

**Keywords:** sleep, incarceration, trauma, healthcare, carceral facilities, prison, prison environment

## Abstract

**Study Background/Objectives:**

Sleep is an underexplored factor in the health of people involved in the criminal legal system. This study addresses the paucity of research on how individual, social, and physical environmental factors impact sleep health during and after incarceration by highlighting the voices of people involved in the criminal legal system through a community-engaged qualitative research approach.

**Methods:**

We conducted 20 semi-structured interviews with men recently released from prison for a study on trauma and healthcare during incarceration and after release. Interviews were coded and analyzed using reflexive thematic analysis and a critical realist framework. Our research team included people with a history of incarceration who performed central roles in the research process.

**Results:**

Three themes emerged from participants’ descriptions of sleep during and after incarceration: (1) concerns about health contributing to sleep problems, (2) lack of access to treatment for sleep disorders leading to ongoing sleep problems, and (3) issues of safety contributing to sleep problems during incarceration and after release.

**Conclusions:**

This study identifies factors and domains influencing sleep during and after incarceration. By identifying which interpersonal, environmental, and structural factors impact sleep quality, medical and carceral staff are better equipped to ameliorate sleep health disparities within populations with a history of incarceration and those actively bound by the criminal legal system. Future research should examine other factors impacting sleep in incarcerated and recently released populations and develop multi-level interventions to improve sleep health.

**Statement of Significance**

Sleep is an underexplored factor in the health of people involved in the criminal legal system. Incarcerated people are susceptible to unique individual, environmental, and institutional/policy-level factors that may negatively impact sleep during and after incarceration. Incarceration may contribute to sleep health disparities in minoritized communities given the disproportionate incarceration of racial and ethnic minoritized groups. The current study aims to identify the mechanisms perpetuating poor sleep in carceral settings, expanding on literature that identifies poor sleep in the carceral environment and incorporating people with a history of incarceration as co-researchers.

## Background

Constituting almost one-third of our lives, sleep is essential for human survival [1]. This routine altered state of consciousness serves a number of important health functions from memory consolidation to maintaining overall health and wellbeing [2]. Poor sleep health is marked by deficiencies in quantity and/or quality of sleep, resulting in suboptimal health [3]. Varying levels and manifestations of poor or deficient sleep (insomnia, parasomnia, insufficient sleep duration, sleep apnea, etc.) affect 50–70 million people in the United States, with disproportionate morbidity in low-income and racial and ethnic minoritized populations. The causes of poor sleep health are multifactorial and multi-level, spanning mental health conditions (e.g. depression, post-traumatic stress disorder) to social and physical characteristics of the sleep environment [4–7].

Neighborhoods characterized by greater social disorder, lower social cohesion, and lower safety are associated with shorter sleep duration, even after controlling for resident socioeconomic status and physical environment [8–11]. A study on social patterning of sleep in African Americans by Johnson et al. showed low education and low income’s association with long sleep (>9 h) and poorer sleep quality, and high neighborhood violence’s association with shorter and poorer quality sleep [12]. Conversely, positive aspects of the physical neighborhood environment, such as lower ambient noise levels, are associated with improved sleep [13, 14]. Similarly, self-reported neighborhood safety has been associated with lower daytime sleepiness [15]. A recent review showed promising improvements in sleep health by addressing environmental characteristics [7], and emerging research identified sleep environment as an important modifiable mediator between poor sleep and cardiovascular disease risk factor (hypertension, diabetes, obesity) management [5]. If your “neighborhood” is a carceral facility, your sleep may be suboptimal. Incarceration, however, is a virtually unexplored factor contributing to poor sleep health despite its prevalence in the United States and its disproportionate impact on minoritized populations.

Over two million people are incarcerated in the United States, and an estimated 11 million individuals cycle through jails and prisons yearly [16–18]. Minoritized groups are disproportionately incarcerated with Black people incarcerated at five times and Hispanic people at 2.5 times the rate of White people [19]. Incarcerated people may have unique individual-level reasons for poor sleep health. For instance, incarcerated people globally have higher rates of mood disorders and chronic pain, which amplify poor sleep, compared with those who have never been incarcerated [20–22]. Carceral systems’ social and physical environment may also impact sleep. Exposure to violence and stressful interpersonal relationships between incarcerated people and with staff are associated with increased psychosocial stress and sleep problems (e.g. nightmares, insomnia) [3, 7, 10, 23, 24]. Environmental factors like noise, light, air quality, and extreme temperatures similarly contribute to poor sleep [25–29]. Restrictive policies around sleep/wake schedules, lockdowns, and overcrowding in carceral facilities compound these negative impacts [25, 26, 30]. Carceral systems often use sleep deprivation as a form of control through “health checks” (read: constant forced waking), 2 am med calls, and night-shift work [4, 30, 31]. In our clinical, research, and lived experience, extant sleep issues are further exacerbated by an overt lack of access to assessment for sleep problems, over-the-counter sleep aids (e.g. melatonin), and standard sleep treatments (e.g. prescription sleep medication, CBT-I). In response to sleep complaints, carceral medical staff often prescribe sedative psychiatric medication. Incarcerated people may take matters into their own hands and use illicit drugs as sleep aids. For others, sleep problems go untreated. Assuming they are even able to acquire healthcare after release, people are often unable to get prescriptions for the medications they received while incarcerated because providers think the medications prescribed are no longer appropriate.

Following release, sleep environments may be unstable or remain under supervision of the criminal legal system. People returning to the community live in various settings (e.g. with family/friends, in shelters or halfway houses). For some, moving from a highly regimented and controlled environment to one with little structure can prove challenging. Others experience the move to congregate housing, shelters, or halfway houses as a continuation of the carceral environment, still governed by restrictions on when one can eat, work, sleep, what medications one can receive for sleep disorders, and contentious interactions with staff and roommates. In both cases, those returning to the community often live in low socioeconomic status neighborhoods, a predictor of waking after sleep onset [23].

Despite the increased risk for poor sleep health during incarceration and following release, there has been limited research conducted in the United States on the sleep implications of incarceration and almost none that focuses on minoritized populations [32, 33]. Past studies are primarily quantitative and focused on insomnia in carceral settings with inconsistent results, varying from 11% to 81% morbidity [27, 32, 34–41]. While a number of studies describe light, noise and exposure to violence in their background sections as environmental factors that potentially influence sleep, no studies have provided comprehensive description of sleep environments experienced by incarcerated people or those just released [38, 42–44]. The current study centers the voices and lived experiences of incarcerated persons’ to understand how individual, social, and physical environmental factors contribute to sleep problems during incarceration and after release.

## Methods

### Study Overview

We conducted and analyzed 20 semi-structured qualitative interviews with men recently released from a carceral facility focused on healthcare experiences and trauma during incarceration [45]. The interview guide was designed, tested, and revised with input from the study PI, staff with a history of incarceration, and medical students. While sleep was not explicitly asked about, it nonetheless emerged as a significant motif in our original coding. The current analysis focuses on that emergent sleep theme. We extracted all sections of interviews in Dedoose (a qualitative data management software) coded for sleep and conducted thematic analysis using an inductive/descriptive approach and critical realist framework. This approach ensured that the analysis was focused on the voices of the participants and how they thought and felt about sleep [46–51].

This study was approved by the Yale University Institutional Review Board. We report our study results using the Consolidated Criteria for Reporting Qualitative Research (COREQ) guidelines for qualitative research [52]. Additional information on the original study methodology is detailed in the initial publication of results [45].

### Sampling

We recruited 20 men recently released from a carceral facility between 2018 and 2019 using a purposive sampling strategy. Most participants were released in the last three months, and all were released less than one year prior to the interview improving clear recall of their incarceration experience. Recruitment largely took place through a community-based primary care program for people with a history of incarceration located in New Haven, CT, where patients received information about the study and were asked to contact the research team if they were interested in participating. Only one person did not participate in the study because of serious health issues. Some participants previously met the research team through their connections to the clinic or because some of the interviewers had a history of incarceration. To address this, we paired participants with interviewers with whom they did not have a professional relationship or history as acquaintances. Participants were introduced to the interviewers and were aware of their history of incarceration and/or experience and background and the purpose of the study. Interviews were 60-90 minutes, and participants were paid $30 for their time. The interviewers checked in with participants at the end of the interview and were prepared to refer them to services if further help was needed. All participants were given a sheet with local resources that included information about accessing basic needs and medical and behavioral healthcare. Saturation was reached prior to the twentieth interview, but a few additional interviews were done to ensure saturation.

### Data generation

Our team developed a semi-structured interview guide that was tested in mock interviews before being used in the study. All study activities were conducted in private offices, and only participants and the interviewers were present in the room. All interviews were preceded by the consent process, followed by a brief survey focused on participants’ mental health and incarceration history. The interviews were conducted by pairs that included the study PI and either a research assistant with a history of incarceration (number of interviews conducted: 19/20) or a medical student (number of interviews conducted: 1/20). Interview guide questions focused on the men’s experiences of medical care and trauma during incarceration and after release and did not ask about sleep. Each participant was interviewed once. Interviews were recorded using audio recorders and then transcribed using Rev.com. The transcripts were reviewed for accuracy and uploaded into Dedoose for analysis.

### Data analysis

We used an inductive and descriptive approach to reflexive thematic analysis and a critical realist framework to center the voices of the men we interviewed and their perspectives on the carceral experience [46–50, 53]. This approach employed an empathetic interpretation of participants’ subjective perspectives on the carceral experience, requiring the researcher to interpret the interviews from the participant’s perspective and consider the social and cultural context in prison and after release [46–51, 53]. Our analysis team included the study PI, a medical student, and a student intern and research assistant, both with a history of incarceration. Additional input on the analysis was also provided (phases 5 and 6) by the two MDs with expertise in incarceration and health and sleep medicine. Table 1 shows the steps in the analysis process and which team members were involved in each step.

**Table 1.** Steps of reflexive thematic analysis

Phase of analysis	Process	Team member involvement
Pre-analysis	Transcripts reviewed for accuracy and errors corrected.	PI Medical student with experience working with people with a history of incarceration in community and healthcare settings
Phase 1: familiarization	Listening to recordings and reviewing transcripts.	PI Medical student with experience working with people with a history of incarceration in community and healthcare settings
Phase 2: code generation	Codes generated and refined. Revisited the transcripts to ensure that all sections where sleep was discussed were identified and any additional sections were coded for sleep.	PI Medical student with experience working with people with a history of incarceration in community and healthcare settings
Phase 3: theme generation	All sections of the interviews coded for sleep were reviewed.	PI Intern with a history of incarceration
Phase 4: review and refine themes	Initial themes were generated and then reviewed several times by the team and discussed to refine the themes.	PI Intern with a history of incarceration Research assistant with a history of incarceration
Phase 5: defining and naming of themes	The themes were defined by the analysis team and named.	PI Intern with a history of incarceration Research assistant with a history of incarceration MDs with expertise in incarceration and health and in sleep medicine
Phase 6: report	The iterative process of refining the themes and their connections continued through the reporting process.	PI Intern with a history of incarceration Research assistant with a history of incarceration MDs with expertise in incarceration and health and in sleep medicine

The analysis was inductive and began with familiarizing ourselves with the transcripts by first reading through them without assigning any codes. Codes were identified during the analysis and were not developed in advance. Initial codes were produced, reviewed, discussed, and revised by the coding team. For the sleep analysis, we then identified all sections coded “sleep” and reviewed and analyzed sleep-related quotes to identify patterns in the codes and new emerging themes. Since the original study was not focused on sleep, analysis was limited to quotes related to sleep, and no minor themes related to sleep were identified in the analysis. To ensure we did not miss any key quotes related to sleep, we also went back and searched the transcripts for words related to sleep to confirm that we did not miss anything in the initial coding. While participants did not review the coding or final analysis for this project, team members with a history of incarceration were part of the coding and analysis team. They contributed to and reviewed the analysis of the study data and this manuscript. All participants were assigned pseudonyms to protect their privacy, given the sensitive nature of the interviews.

### Engaging people with a history of incarceration in research

Central to this project and all our work is including people with a history of incarceration and other key stakeholders in the health of people who have been incarcerated (e.g. community health workers, community, and carceral health providers) as partners in the design, data collection, analysis, and dissemination of our research. While many community engaged research projects connect with community-based organizations for expertise around a specific population, we sought to engage men with a history of incarceration as co-researchers. This is particularly important in research with populations with a history of harm and distrust of the systems they are a part of, in this case, carceral and healthcare systems. Two men with a history of incarceration were hired as research assistants for the project, and two medical students participated as interviewers and as part of the coding team. As a group, they received training in qualitative research and interviewing. In collaboration with the PI, the men with a history of incarceration developed the interview guide and conducted most interviews. This served several purposes, including building trust with the study participants, who were asked to share their very personal experiences of healthcare and trauma during incarceration. They could see themselves in the interviewers, and their shared experience allowed them to talk openly about the things that happened while they were incarcerated and after release.

The design, implementation, and data analysis of this project were informed by the authors’ lived experience of incarceration and their experience in providing medical care and conducting research about people with a history of incarceration. We aimed to have people from diverse backgrounds and perspectives at each stage of the research to ensure that we were considering a variety of factors and not including only one viewpoint in the study. Past research has shown that without the input and leadership of people directly impacted by incarceration, interventions are not adopted by correctional and healthcare systems, utilized by patients, or disseminated to scale [54, 55].

## Results

Study participants spent an average of 11 years in prison and were incarcerated 6.4 times. Participants included Black (40%), White (20%), and Hispanic and Latino (40%) men with a mean age of 46 (range, 23–58 years). Almost all participants (95%) had spent time in solitary confinement (meaning they were isolated from other incarcerated people and staff and spent 23 hours a day alone in a small cell), ranging from 14 days to 15 years. Almost half of the participants (45%) had been diagnosed with post-traumatic stress disorder. Table 2 describes the demographics of the study sample.

**Table 2.** Participant characteristics (*n* = 20)

Race/ethnicity Black White Hispanic and Latino	8 (40) 4 (20) 8 (40)
Age (years) Mean Range	46 23-58
Length of most recent incarceration Mean Range	11 years 2 months-30 years
Solitary confinement	19 (95)
Posttraumatic stress disorder (PTSD)	9 (45)

Data are reported as *n* (%) unless otherwise specified.

Participants consistently described their struggle to sleep, primarily related to fear connected to various aspects of their incarceration experience. Three themes characterized participants’ sleep experiences during and after incarceration:

1. Concerns about health contributing to sleep problems
2. Lack of access to treatment for sleep disorders leading to ongoing sleep problems
3. Issues of safety contributing to sleep problems during incarceration and after release

### Theme #1: concerns about health contributing to sleep problems

Study participants indicated that (lack of) healthcare interfered with their sleep in two primary ways: (1) limited or poor care created anxiety that affected sleep, (2) concerns about health disrupted sleep, especially worrying that they might die in their sleep. Several men talked about how fear of dying contributed to sleep problems through hypervigilance or perseveration around their health. Participant’s lack of trust in the medical staff often led to concerns about the severity of the health issues they were presenting to the carceral facility medical providers.

**You get scared**, I’m not going lie. You really do get scared because you never know if one day you might go through something that’s **life-threatening** and they have no solution for you. So, it’s like now your down at medical and you’re feeling like you’re damn near on your **deathbed,** and it’s like, the only thing they can tell you is, “Oh you’ll be all right, here’s some Motrin.” Or, “You’ll be all right, **just sleep it off** and come see us tomorrow.” So now it’s like, what if you do go back, and **that’s your last sleep**? (Melvin)^1^

Thoughts like this can lead to short-term sleep disturbances or longer-lasting issues like insomnia, which continue even after release from prison without intervention. Here, as in many of our interviews, fear and anxiety about their health prevent the person from getting restful sleep.

Well, he—unbeknownst to everybody, he had drilled in and severed an artery, and I was bleeding and bleeding and bleeding.… Ah, and **I was starting to worry and couldn’t really go to sleep**, and I nodded off at one point, like 3:00 in the morning, just for a couple seconds. Uh, you know, I had the gauze in there and-and **I woke up startled**, **and by then, the blood was everywhere**. It was-and I called the guard over to the door and he was like, “Nothing we can do; it’s 3:00 in the morning.” **So I’m basically bleeding out**. And, uh, If I had gone to bed normally, I would have, you know--wouldn’t know, that the gauze would have come loose, and I would have just, you know, if I didn’t choke I would have, you know, I would have just bled out. (John)

The concerns expressed by both participants highlights an apprehension that all of the men expressed around worries about health and the distress that they caused. Our earlier work from this study [45], describes in detail the trauma the men experienced as a result of the healthcare they received in these carceral facilities. Illness and pain from health problems or injuries were routinely brought up by the men interviewed. In carceral settings, concerns about trust and privacy may additionally prevent people from seeking care and contribute to sleep problems.

### Theme #2: lack of access to treatment for sleep disorders leading to ongoing sleep problems

More than just concerns about health, lack of treatment for sleep problems negatively impacted sleep health during incarceration and after release. Many of the men described what they had to do to ensure their safety and that this often had the unintended consequence of making them a potential target for retaliation.

But, sometimes it takes…I might have to hurt five people for you to understand that I’m not that person, to leave me alone. But, what’s, you know, **it comes with damage, and it also comes with the fear because now I got to watch my back**, I got to turn around, I got to do all of that. **So, it causes you not to sleep at night, and then you can’t get medication for that. So, you can’t get a sleeping pill, so you turn to drugs**. (Darnell)

Participants described being prescribed psychotropic medications that they could not access after release leading to worse sleep. Others talked about using illicit drugs to help them sleep when they could not get help with their sleep from the medical staff. For some, using suboxone not prescribed for them or illicit drugs was the only option when they could not get the medical staff to prescribe something to help with sleep. Conversely, access to treatment for trauma (e.g. from enacting, being victim to, or witnessing violence) and sleep medications upon release affected participants’ abilities to sleep and limit or eliminate nightmare occurrences.

When I was incarcerated, **I was having a lot of nightmares and flashbacks** and, um....happened to me in my past. I have had, um, bunkies, tell me that, **“When you sleep, you talking in your sleep. You’re real loud in your dreams and, you know, you [have] vivid dreams of this crazy stuff”**. And I’m like, yeah. This is just, you know, things that I been through. I thank God I’m on my medications now. You know, I’m dealing with it better. I’ve been on several sleep meds. The more I stay consistent, the better I am, you know? (Peter)

Sleep problems that develop during incarceration, often continue after release. A few participants noted that parasomnias, both Rapid Eye Movement (REM) (e.g. RSBD, nightmare disorder, sleep paralysis) and non-REM (e.g. sleepwalking, night terrors), impacted their sleep during incarceration and commented on their inability to get treatment until after release.

Some described Rapid Eye Movement (REM) Sleep Behavior Disorder (RSBD) and somnambulism (sleepwalking) that went untreated and continued after release. RSBD involves the acting out of dreams when the body should be paralyzed to prevent movement that could cause injury to the self and others. These types of sleep disorders can cause problems in congregate settings and lead to concerns about safety and sleep deprivation in their attempts to avoid issues with the staff or other residents.

“You did it, you’ve done it three times since you’ve been here.” You know? She says, “A couple times you actually fell.” I don’t remember that. You know what I mean? So now they’re saying that **I’m sleepwalking**. You know. **Now I’m freaking out about that**. You know what I mean? **So, I barely sleep**. Now I’m, now **I’m freaking, these uh... warming centers and I can’t sleep**. So I’m worn out. Worried….I know**, a similar thing happened when I was in prison**. I was in there so much. I was in there for so many years, that, I used to walk to breakfast, and then I used to wake up in the breakfast, sit down, and actually wake up. (Miguel)

Participants noted feeling like the medical staff made assumptions about their requests for help with sleep being linked to drug-seeking behavior.

### Theme #3: issues of safety contributing to sleep problems during incarceration and after release

Most participants discussed the impact of violence and deaths in the facilities as causing sleep disruption, rumination, and nightmares.

I was like twenty-three at the time or something like that, and he was like twenty-five and **I was sleeping, I just heard all this racket**. This noise, yelling, I’m like, “What in the world is going on?” So, I come to my cell and I look out the door and **I see my friend on the floor, they’re trying to resuscitate him. I’m like, “What the-?” And he died**, and I was like, “Yo that’s crazy.” I mean, it, it destroyed me, because that was my best friend and the first thing came to my mind is like, “I can’t die in here. He’s twenty-five years old. I can’t, I can’t do it. I’m two years younger than him. (Robert)

Participants often ascribed more severe violence to sleep problems, including insomnia, hypersomnia, nightmares, and night terrors. Violence exposure may also contribute to difficulty achieving the appropriate duration for each sleep stage, particularly rapid eye movement (REM) sleep.

**I saw a couple people get stabbed, um, real badly, and sometimes I wake up in my sleep thinking about it**. And um... seeing - seeing one person get killed, um, one person jumps off of the tier and hung himself... because his needs wasn’t being met and, um... yeah a lot of things I saw is still kinda like, um, affects me a little bit. Like **sometimes I wake up out my sleep, and I think about it**. (Carl)

Sleep was not a time of rest and ease for these men. Assaults could come from cellmates, other incarcerated men, or carceral officers. Some took precautions when they went to bed, keeping a weapon ready or sleeping lightly, both in an attempt to be ready for violence during the night.

I’m so used to so many years always putting my back to the wall that way, somebody don’t sneak up and try to stab me or nothing. I ain’t gonna lie to you, I, I kept a knife til, til the night before I went home**. That knife was like my girlfriend, when I went to sleep I just put it right next to my bed and go to sleep**. (Ralph)

Many participants described being light sleepers, able to wake up to any sign of noise that might predict danger, indicating that they might not get to the deeper stages of N3 and REM sleep necessary for cell recovery, memory consolidation, and overall good health.

Some talked about a disregard for those who were trying to sleep and the decisions they had to make about whether to say something or to stay quiet and go without sleep. We going to make some wine and we going to sit around and drink and reminisce and talk about something. And then when this guy over here say something, **we just beat him up, because he wanted to go to sleep**; it’s 11 o’clock at night, and you want that. So to deal with all of this stuff that we not dealing with, you lash out, and you hurt somebody else (Darnell)

Several participants also talked about how the prior carceral environment, untreated sleep conditions, and the environment and stress of participants’ lives after release affected their sleep when they returned to the community. Participants described how violence in prison affected their interactions with others and sleep after release. Congregate settings like halfway houses and shelters increased social stress for several participants.

That made me real antisocial. I’m not gonna lie. Like even out here, like. When I was in the halfway house, **I actually made a little like cell for myself. I put towels all around my bunk just so I could be alone**. (Jay)

While some had trouble sleeping because they were living in an unstable environment after release, even those living in safe places reported lingering concerns about safety due to their experiences during incarceration.

And **I’m scared, and that’s the truth…I don’t sleep in the bed. I have a bed, but I sleep on the couch because it’s next to the front door.** My daughter’s like, “Dad, sleep upstairs.” And I’m like, “No, I’m good. I wanna watch TV.” But I don’t tell her that. “I’m all right.” …But, I’m more comfortable sleeping on the couch where I know where the door is and I can hear it open. **It don’t matter if I’m sound asleep or not, if the door opens, I’m up**. (Darnell)

Several of the men who were plagued by parasomnias experienced vivid dreams about the violence they witnessed or experienced while incarcerated.

It stays at a high level and constantly like that and you worry and then, like I said, with the-the all the physical conditions weren’t that bad, the psychological conditions it really wears on you. It just, day after day, year after year…..**I went to bed early and uh, in my dreams were there**, I mean little things would aggravate me and I would have these uh, vivid uh, dreams, it was. **My dreams, I mean for decades now have been either, um, someone’s trying to kill me or I’m trying to kill them, either in or out of prison.** And you know, between all the inmate on inmate violence and staff on inmate violence, and inmate violence on staff. There was different variants of that in the dreams. And I could get up in the middle of the night to go to the bathroom whatever, and go right back to the same dream. You know. You know. **And the real intense ones, sometimes I’d wake up in a cold sweat and panting** and you know- **The whole thing, feeling like I’d just been there, you know, like I experienced it**. (Glenn)

The voices of this group of formerly incarcerated men highlight the ways that trauma, caused by exposure to violence or healthcare experiences, can impact sleep in the moment and long after those experiences have ended. These quotes offer a glimpse into the lives of this group of formerly incarcerated men and illustrate the many ways that living in a carceral facility can have both short and long-term impacts on sleep health.

## Discussion

This study explores how spending time in carceral facilities creates sleep problems during incarceration that can persist after release and negatively impact overall sleep health. We describe some of the primary factors incarcerated people felt interfered with their sleep. Participants identified both healthcare and violence as key drivers of sleep problems and voiced three primary themes: concerns about health contributing to sleep problems; lack of access to treatment for sleep disorders leading to ongoing sleep problems; issues of safety contributing to sleep problems during incarceration and after release.

Our results identify some ways the treatment of health conditions in carceral facilities can contribute to sleep problems and our earlier paper from these interviews highlights healthcare-induced trauma in carceral facilities [45]. A previous study of insomnia in incarcerated people found that those with insomnia were more likely to have a concurrent medical or mental health condition [56]. Concerns about health and the paucity of treatment options during incarceration, not just the health conditions themselves, contributed to insomnia and should be investigated in future studies.

Few studies on sleep and incarceration have focused on what assessments, treatments, and interventions for sleep problems are available for this population. Medications commonly used to treat sleep in carceral settings include over-the-counter remedies like Benadryl or allergy medications and psychotropic medications like Remeron (anti-depressant) and Seroquel (antipsychotic) [43]. These medications may be used in lieu of insomnia medications used in community settings (e.g. Ambien, Lunesta, Belsomra) because the common causes of lack of sleep in carceral settings (anxiety, trauma, fear of violence) may be viewed as needing a stronger sedative to counteract. The use of these medications during incarceration may help sleep in the short term, but cause problems after release. Participants reported that they were often unable to get these medications from their primary care provider in the community, contributing to worse sleep after release. Noted by participants in this study and in our clinical, research, and lived experience, lack of access to sleep medication and CBT-I—options that are readily available to people outside the carceral system—exacerbates sleep problems, leading to mood changes that negatively moderate interactions with cellmates, other incarcerated people, and facility staff. Given the interconnected nature of trauma and trauma-related sleep problems, integrating CBT-I with PTSD treatment may improve the efficacy of sleep-related care in this population. Access to formal assessment and treatment for sleep disorders and comorbid mental health conditions during incarceration and after release could improve sleep health and reduce exacerbation and development of other health problems, improving mental health and social climate in facilities.

One study explored the use of interventions like mindfulness to improve well-being, using sleep as a secondary outcome, finding mindfulness-based emotional intelligence interventions decreased anxiety and depression and significantly improved sleep quality scores on the Pittsburgh Sleep Quality Index (PSQI) following intervention [42]. While CBT-I is commonly used to treat insomnia in community settings, it is not a treatment available in carceral settings despite its proven efficacy in this population. A study by Randall et al. [32] showed promising results using a one-shot CBT-I intervention to treat insomnia in incarcerated men, showing a reduction in the severity of insomnia and a reduction in depression and anxiety. A better understanding of the variety of sleep problems faced by incarcerated people and the factors contributing to sleep disparities in this population will aid in the adaptation and development of more population-specific interventions. Furthermore, a bolstered preventative care structure in carceral settings can reduce urgent care load and proactively improve sleep health.

Rumination and nightmares were barriers to sleep, triggered by fear of violence in the carceral social environment. In carceral facilities, noise and violence often go together, with noise signaling events that could be disturbing to witness or directly experience. Even requesting others to be quieter can incite violence. One must choose between sleep and surviving the night. These coping tactics create a positive feedback loop in which sleep deprivation due to fear of violence propagates more violence. Lack of sleep has been shown to increase sensitivity to even minor events, resulting in inappropriately volatile retaliations [57].

While here we focused primarily on how healthcare and trauma impact sleep, some participants did mention how factors in the physical environment can contribute to sleep problems. Adornetti et al. [58] highlight the impact of light and facility schedules on the sleep of juveniles in detention: issues in both juvenile and adult facilities that could be addressed to improve sleep. A recent review indicated the promise of addressing environmental characteristics to improve sleep, and emerging research identified sleep environment as an important modifiable mediator between sleep deficiency and worsening management of cardiovascular disease risk factors, including hypertension, diabetes, and obesity [5, 7]. Future studies should also examine how the physical environment in correctional facilities can affect sleep health.

While past research has pointed to noise, light, facility elements, and sleeping conditions as potential causes of sleep problems in this population [25–27, 31, 59], no studies have examined sleep after release from prison. The possible mechanisms of poor sleep health during incarceration and after release should be explored in future work to better understand and mitigate racial disparities in sleep health.

### Strengths

Key strengths of this study include the centering of voices of formerly incarcerated people through their inclusion as co-researchers on the team and the use of qualitative methodology to understand lived experiences of incarceration. The interviews were enriched because participants trusted the interviewers, primarily because they also had a history of incarceration and/or direct experiences of the impact of incarceration. This was particularly important because most of the participants served long prison sentences, and building trust was essential.

## Limitations

The study was limited because the original study was focused on trauma and healthcare during incarceration and did not focus on sleep. Because of the focus on trauma and healthcare, most of what the men describe about their sleep was related to trauma or their healthcare experience during incarceration. It is notable that sleep came up in the interviews even though it was not explicitly asked about, demonstrating the importance and impact of sleep issues for the men in the study. The small purposive sample is a limitation and may increase bias. Since most of the participants had served lengthy prison sentences and experienced considerable trauma before and during incarceration, the presented experiences may be skewed. This study did not include any subjective or objective sleep measures that could provide further evidence of participants’ sleep problems during incarceration. Nonetheless, this study provided valuable insights into people’s sleep in carceral settings. The experiences and themes highlighted here reveal areas of focus for future study and multi-level interventions to improve the sleep health of people involved in the criminal legal system.

## Conclusion

Although sleep is considered a basic need, it is rarely considered in research or programs focused on those impacted by the criminal legal system [60]. We identified new domains that may influence the sleep health of the millions of people with a history of incarceration. This work informs new understandings of how we must address sleep health disparities. Future work should focus on exploring sleep health more broadly among incarcerated people by conducting additional qualitative studies focused on sleep health in this population, studies to adapt existing sleep instruments to better assess sleep in this population, and larger studies that employ subjective and objective measures of sleep both during incarceration and after release. Explorations of sleep during incarceration focused on adapting and testing interventions for sleep and considering multi-level interventions that address sleep at the individual and carceral facility level (e.g. schedule, noise, light) are critical next steps in better understanding and addressing sleep in this population.

### Reflexivity statement

The study PI and her team are based at the SEICHE Center for Health and Justice, a center at the Yale School of Medicine focused on understanding and addressing the public health harms of mass incarceration through clinical care, research, education, and legal scholarship and advocacy. Our research team brought diverse perspectives to the study. It included the study’s PI, a social worker scientist, research staff with past histories of incarceration, and medical students. All team members received training in human subjects research and qualitative methods in preparation for the study. Author 1 is a Black female (MSW, PhD) social worker and researcher with expertise in community-engaged research approaches and qualitative research. Author 1 was an Associate Research Scientist at the time of data collection and is currently an Assistant Professor. Author 2 is a queer Asian American male with a BA in Black Studies. They have extensive experience in qualitative methods and are currently working as a postgraduate associate at the SEICHE Center for Health and Justice. Author 3 is a Black male research assistant with a history of incarceration who helped develop the interview guide, conducted qualitative interviews, and contributed to the analysis. Author 4 is a Latina female with an MA in Public Policy and a history of incarceration. She was a student intern at the SEICHE Center at the time of the analysis and is now a law student. Author 5 is an Asian female MD who is a professor of medicine and public health with expertise in incarceration and health outcomes. Author 6 is a White male MD professor of internal medicine with expertise in pulmonary, critical care, and sleep medicine. Authors 1 and 3 developed the interview guide along with other members of the research team and conducted most of the interviews. The two medical students who were part of the research team were white females and had experience working with people with a history of incarceration in clinical settings. The design, implementation, and data analysis of this project were informed by the authors’ lived experience of incarceration and their experience in providing medical care to people with a history of incarceration.

## Supplementary Material

zpad055_suppl_Supplementary_Data

## Data Availability

The interview data used and/or analyzed during the current study are not publicly available due to the sensitive nature of the interviews.

## Footnotes

1 All names used are pseudonyms to ensure anonymity.

## Notes

*This paper is part of the Sleep and Circadian Health in the Justice System Collection.*
